# Crows recognize geometric regularity

**DOI:** 10.1126/sciadv.adt3718

**Published:** 2025-04-11

**Authors:** Philipp Schmidbauer, Madita Hahn, Andreas Nieder

**Affiliations:** Animal Physiology Unit, Institute of Neurobiology, University of Tübingen, 72076 Tübingen, Germany.

## Abstract

The perception of geometric regularity in shapes, a form of elementary Euclidean geometry, is a fundamental mathematical intuition in humans. We demonstrate this geometric understanding in an animal, the carrion crow. Crows were trained to detect a visually distinct intruder shape among six concurrent arbitrary shapes. The crows were able to immediately apply this intruder concept to quadrilaterals, identifying the one that exhibited differing geometric properties compared to the others in the set. The crows exhibited a geometric regularity effect, showing better performance with shapes featuring right angles, parallel lines, or symmetry over more irregular shapes. This performance advantage did not require learning. Our findings suggest that geometric intuitions are not specific to humans but are deeply rooted in biological evolution.

## Teaser

Crows spontaneously identify intruder quadrilaterals among arbitrary shapes, excelling with regular shapes over irregular ones.

## INTRODUCTION

Animals have rudimentary forms of mathematical capabilities. Through their intuitive “sense of number,” which they share with humans, they can estimate and process the number of objects in a set (*1*–*3*). This ability falls under arithmetic, the branch of mathematics that deals with numbers and their basic operations.

In contrast, when it comes to elementary geometry, also known as Euclidean geometry—the branch of mathematics focused on the properties and relationships of basic geometric shapes, such as points, lines, angles, and polygons—the current literature portrays a notable gap between human and animal skills. In humans, geometric intuitions are omnipresent; they develop early in ontogeny (*4*–*7*), are observed across different cultures and irrespective of formal education (*8*–*11*), and have been pervasive throughout human history (*12*).

This is different in animals. While animals from diverse taxa are known to use spatial relationships between landmarks and the surfaces of areas to navigate and to orient in space (*13*–*16*), Euclidean geometry provides a mathematical framework for understanding shapes, objects, and spaces, and is rooted in geometric rules. Animals’ sensitivity to geometric regularity has been found to be notably limited (*17*); nonhuman primates do not recognize geometric regularity in tests involving the perception of visual shapes, whereas humans do (*10*). This result led to the interpretation that the recognition of geometric regularity could constitute a uniquely human ability (*18*).

Here, we examined geometric primitives in carrion crows, corvid songbirds known for their advanced cognitive (*19*–*21*), and arithmetic capabilities (*22*–*24*). We tested how crows perceive visual shapes—particularly quadrilaterals such as squares, rectangles, and parallelograms—using a methodology previously used to explore geometric intuitions in humans and monkeys (*7*, *8*, *10*). If crows can spontaneously discern key geometric properties such as length, parallelism, perpendicularity, and symmetry, then they should perceive regular shapes such as squares more accurately than irregular shapes.

## RESULTS

**Fig. 1.**
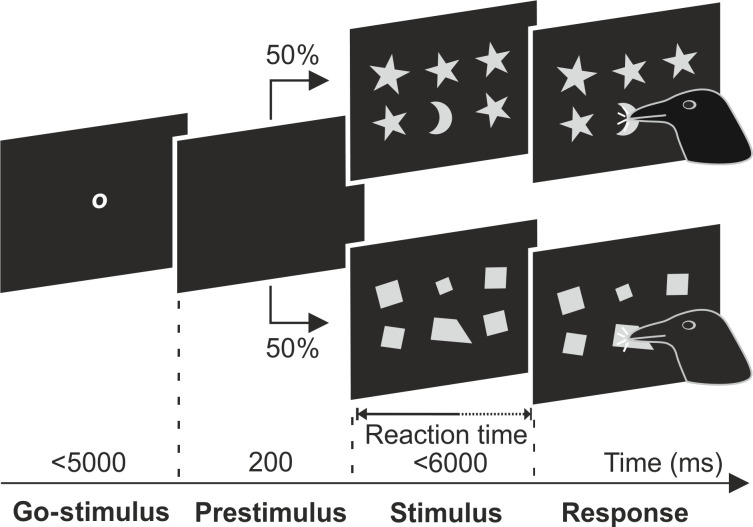
Intruder detection task. Two carrion crows were trained to detect an intruder stimulus in an array of six simultaneously presented stimuli. The crows initiated the trial by moving their head in front of the screen whenever a go-stimulus appeared. After a prestimulus period of 200 ms, an array of six stimuli was displayed. The crows responded by pecking on the intruder. In 50% of the trials, the crows were tested with non-quadrilateral background stimuli (**top**); in this example, the crescent is the intruder among stars. In the other 50% of the trials, the crows were tested with quadrilateral probe stimuli (**bottom**); in this example, a nonsymmetric quadrilateral is the intruder among symmetric quadrilaterals. Background and probe stimuli appeared pseudo-randomly. All stimuli were individually rotated and scaled in size.

We trained two carrion crows to detect a distinct display among two-dimensional visual shapes (Fig. 1). The crows were presented with a stimulus array of six simultaneously displayed shapes and had to peck on the outlier (the “intruder”) that differed in visual parameters compared to the remaining five base stimuli. Initially, the crows were trained with stimuli differing in shape, pattern, or color. During two generalization phases, the crows successfully applied the general principle of detecting intruders to new stimuli, including simple polygon shapes. The stimulus pairs used during the training and generalization phases are shown in fig. S1; none of the pairs included two quadrilateral shapes.

**Fig. 2.**
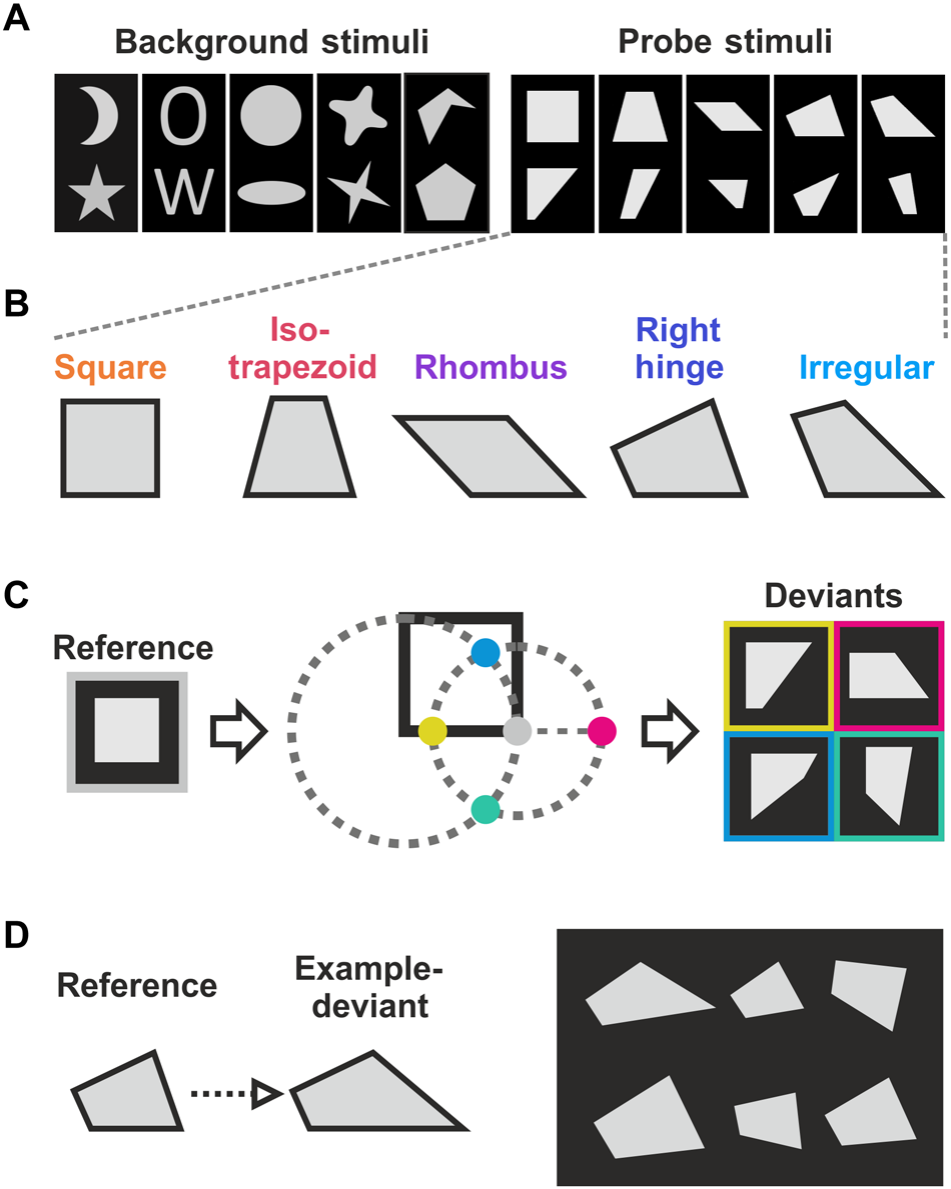
Stimulus set and quadrilaterals. (**A**) Test stimulus set. The crows were tested with a stimulus set consisting of five pairs of nonquadrilateral shapes used as familiar background stimuli that had been used during training (left) and five pairs of quadrilateral shapes used as probe stimuli (right). (**B**) Quadrilateral shapes. The five quadrilateral reference shapes used to test deviation from geometric regularity. From left to right, the quadrilaterals increasingly differed in geometric properties (parallelism, symmetry, perpendicularity, equal sides, and equal angles). Here, they are ordered from left to right by decreasing regularity. (**C**) Construction of deviant probe stimulus shapes. For every quadrilateral reverence shape (left), four deviant shapes (right) were constructed by displacing the bottom right vertex by a fixed distance (center). This resulted in shortening, lengthening, or rotating the bottom side and the right side of the quadrilateral. Within a presentation block, each reference shape was paired with the same deviant shape. (**D**) Example of a canonical probe stimulus layout. Here, a deviant shape (in the top-left position) was placed amid five right hinge reference shapes and had to be recognized as the intruder.

After becoming proficient in detecting intruders, the crows were tested for recognizing geometric regularity with a new stimulus set. This set included five pairs of new quadrilateral reference shapes and their deviant quadrilateral as probe stimuli, along with five pairs of familiar shape stimuli as background stimuli (Fig. 2A). The five new quadrilateral reference shapes varied continuously in their geometric properties, such as the number of parallel sides, symmetries, right angles, equal sides, and equal angles (Fig. 2B and table S1). For each reference quadrilateral, four deviant shapes were created by systematically displacing the bottom right vertex by a fixed distance, thereby altering the length or orientation of the bottom and right side of the quadrilateral (Fig. 2C).

To test the crows’ abstract ability to find the intruder based on geometric regularity difference, the stimulus arrays were presented in two formats: In one format, the deviant shape served as the intruder stimulus, while the reference shapes acted as base stimuli (“canonical” presentation format). An example of a canonical probe stimulus layout is shown in Fig. 2D. In the other format, this arrangement was reversed (“swapped” presentation format). More example probe stimulus layouts are depicted in fig. S2.

**Fig. 3.**
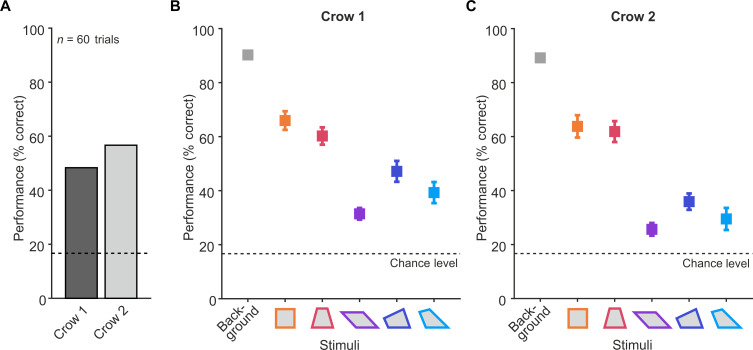
Crows’ intruder detection performance. (**A**) Generalization to novel quadrilateral probe shapes. (**B**) Average detection performance of crow 1 across all presentation blocks (*n* = 35). The average performance across all background stimuli (gray) is displayed together with the average performance for each quadrilateral probe stimulus separately (color). The chance level is indicated by the dashed line. (**C**) Average detection performance of crow 2 across all presentation blocks (*n* = 26). Same layout as in (B).

We hypothesized that if crows were sensitive to the geometric features of quadrilaterals, then they would spontaneously generalize the concept of detecting visual outliers to novel quadrilateral shapes without requiring learning. To investigate this, we analyzed the crows’ performance during trials when they encountered the novel quadrilaterals for the first time. For each of the six positions in the stimulus array, an individually rotated and scaled quadrilateral probe stimulus was generated (Fig. 2B). It is crucial to emphasize that up to this stage of the experiment, the crows had never been tasked with detecting a quadrilateral intruder amid an array of other quadrilaterals. Despite this, both crows were able to spontaneously detect the intruder the first time they were tested with purely quadrilateral shapes. Their performance was significantly above the chance level of 16.7% (one of the six images) within the first 10 trials, which included randomly selected combinations of intruder and base stimuli across all five quadrilateral shapes and two presentation formats. For crow 1, 50% of the probe trials were correct (*P* = 0.015, Cohen’s *h* = 0.73, chance level of 16.7%, one-sided binomial test), and for crow 2, 60% of trials were correct (*P* = 0.002, Cohen’s *h* = 0.93, chance level of 16.7%, one-sided binomial test). The cumulative performance from the first 10 to the first 60 probe trials remained stable and significant (fig. S3). The average performance for the first 60 probe trials—which encompassed all first time presentations of all possible combinations of quadrilateral intruder stimulus, intruder position, and presentation format—is shown in Fig. 3A. Crow 1 demonstrated a detection performance of 48.3% correct, while crow 2 achieved 56.7% correct, both significantly above the chance for the novel quadrilateral shapes (both crows: 60 trials, crow 1, *P* < 0.001, Cohen’s *h* = 0.70; crow 2, *P* < 0.001, Cohen’s *h* = 0.86, chance level of 16.7%, one-sided binomial test).

To gain a detailed understanding of the crows’ spontaneous generalization abilities, we analyzed their correct responses separately for the canonical and swapped presentation formats during their initial exposure to the quadrilateral shapes. Both crows demonstrated the ability to identify the intruder regardless of the presentation format. In the first trials using the canonical presentation format, crow 1 correctly identified the intruder in 33.3% of the trials, while crow 2 achieved this in 66.6% of the trials (both crows: 30 trials, crow 1, *P* = 0.02, Cohen’s *h* = 0.39; crow 2, *P* < 0.001, Cohen’s *h* = 1.07, chance level of 16.7%, one-sided binomial test). For the swapped presentation format, crow 1 achieved a performance of 63.3% accuracy, while crow 2 achieved 46.7% accuracy with the novel quadrilaterals (both crows: 30 trials, crow 1, *P* < 0.001, Cohen’s *h* = 1.00; crow 2, *P* < 0.001, Cohen’s *h* = 0.66, chance level of 16.7%, one-sided binomial test).

Next, we investigated whether the crows’ ability to identify the intruder quadrilateral scaled with its degree of geometric regularity. We calculated the average detection performance for each crow across all blocks, focusing separately on the five quadrilateral shapes. Each block comprised 120 unique combinations of intruder stimuli and positions, where correct identification was required once to progress to the next block. Erroneous trials were reintroduced randomly within the same block. For analysis, we considered only the initial 12 unique presentations per stimulus pair (6 intruder positions × 2 presentation formats) within each block.

Over 10 sessions, crow 1 completed 35 blocks (Fig. 3B), and crow 2 completed 26 blocks (Fig. 3C). Both crows showed average detection performances above chance level for each quadrilateral shape (crow 1: all quadrilaterals ≥ 31.4% correct; crow 2: all quadrilaterals ≥ 25.6% correct), with crow 1 completing 420 trials per quadrilateral and crow 2 completing 312 trials per quadrilateral (crow 1: all *P* < 0.001, Cohen’s *h* > = 0.35; crow 2: all *P* < 0.001, Cohen’s *h* > = 0.22, chance level of 16.7%, one-sided binomial test). The detection performance of both crows differed significantly among the five shapes [crow 1: χ^2^(4) = 51.6, *P* < 0.001; crow 2: χ^2^(4) = 63.9, *P* < 0.001, Friedman test].

We examined whether the observed performance differences among the quadrilaterals could be attributed to their varying levels of geometric regularity. We organized the performances based on the regularity of the quadrilateral shapes, with ease of detection decreasing from left to right as geometric regularity decreased. Both crows showed a decrease in performance as quadrilateral irregularity increased (Fig. 3, B and C). To quantify this trend, a Page’s trend test was used to detect a monotonic trend across ordered levels of expected shape difficulty. We found a significant trend for both crows individually [crow 1: *L* (4) = 1722.5, *P* < 0.001; crow 2: *L* (4) = 1334.5, *P* < 0.001, Page’s trend test]. The crows consistently detected the intruder more effectively when the quadrilaterals were more regular. This indicates that the crows were sensitive to the difference in geometric regularity among the quadrilaterals.

Note that the order of shapes did not strictly adhere to the geometric regularity as quantified by the count of major geometric properties (table S1), but instead followed an empirical regularity scale derived from human subjects (*10*). This resulted in an inversion of positions between the iso-trapezoid and the rhombus (see Fig. 2B). To provide a more conservative assessment of the geometric regularity effect, we repeated the analysis with the shapes ordered according to their mathematically derived geometric regularity. Even in this arrangement, the geometric regularity effect remained significant and consistent [crow 1: *L* (4) = 1652.5, *P* = 0.005; crow 2: *L* (4) = 1271.5, *P* < 0.001, Page’s trend test], indicating that both crows systematically detected the intruder better when the quadrilaterals were geometrically more regular.

**Fig. 4.**
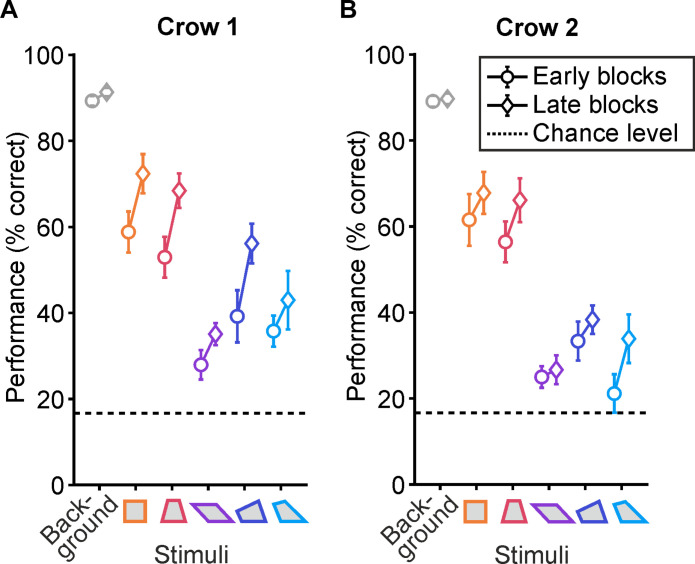
Crows’ intruder detection performance as a function of testing time. (**A**) Average detection performance of crow 1 compared between the early (circle*,* blocks 1 to 17) and the late blocks (diamond*,* blocks 19 to 35). The average performance across all background stimuli (gray) is displayed together with the average performance for each quadrilateral probe stimulus separately (color). The chance level is indicated by the dashed line. (**B**) Average detection performance of crow 2 compared between early (blocks 1 to 13) and late blocks (blocks 14 to 26). Same layout as in (A).

Last, we investigated whether the crows showed improved performance with repeated probe trial repetitions, indicating additional learning effects. We categorized the first half of all probe trial blocks as “early” and the second half as “late” blocks (crow 1: *n* = 17 blocks, crow 2: *n* = 13 blocks per category) and compared the detection performance accordingly (Fig. 4). Both crows showed significant performance already in the early probe trail blocks, with better performances for more regular quadrilaterals [crow 1: *L* (4) = 785.0, *P* < 0.001; crow 2: *L* (4) = 626.0, *P* < 0.001, Page’s trend test]. This pattern of crows showing better performance for more regular quadrilaterals persisted in the late probe trial blocks [crow 1: *L* (4) = 838.0, *P* < 0.001; crow 2: *L* (4) = 604.5, *P* < 0.001, Page’s trend test], albeit with slightly enhanced performance, indicating a learning effect. Consistent with the previously averaged discrimination performance, when the shapes were strictly ordered by their mathematically derived geometric regularity, this geometric regularity effect persisted [early blocks: crow 1: *L* (4) = 756.5, *P* = 0.038; crow 2: *L* (4) = 598.5, *P* < 0.001; late blocks: crow 1: *L* (4) = 802.0, *P* = 0.038; crow 2: *L* (4) = 573.5, *P* = 0.030, Page’s trend test].

A direct comparison between the average performances of early and the late blocks within the same quadrilateral showed marginal improvement over successive presentations of the quadrilaterals. Crow 1’s detection performance did not significantly change between early and late blocks for three out of five quadrilaterals (*P* > 0.05, Mann-Whitney *U* test) (Fig. 4A). However, crow 1 improved in the late blocks for the iso-trapezoid (*U* = 240.5, *P* = 0.018, *r* = −0.41, Mann-Whitney *U* test) and the right hinge (*U* = 248.5, *P* = 0.037, *r* = −0.36*,* Mann-Whitney *U* test). Crow 2’s detection performance did not change over time for any of the quadrilateral shapes (*P* > 0.05, Mann-Whitney *U* test) (Fig. 4B). The detailed probe trial performances per blocks and sessions are displayed in fig. S4. This suggests that the geometric regularity effect was not acquired through learning but was present from the beginning of the test phase. In addition, regularity effect was accompanied by a slight overall improvement in performance across all quadrilaterals.

## DISCUSSION

Our results, showing that crows spontaneously recognize geometric regularity in visual shapes, contrast with those from a study involving monkeys that failed to discriminate quadrilateral stimuli based on geometric regularity (*10*). With systematic improvement in detecting more regular quadrilaterals, crows exhibited a pattern of sensitivity to geometric properties such as parallel sides, symmetries, and angles that resembled that observed in human subjects, albeit with lower overall performance than humans (*10*). The crows’ poorer performance on the rhombus, despite its intermediate geometric regularity compared to other quadrilaterals, mirrors the high error rates observed in adult Himba individuals for the same shape [see (*10*) and Fig. 1H]. This suggests that both crows and Himba participants may prioritize a feature of the rhombus that is less sensitive to variations in the deviant quadrilaterals, resulting in a smaller perceived difference—one not adequately captured by our regularity measurement.

Two methodological differences between our crow study and the study with baboons (*10*) are notable. First, while baboons achieved an 80% correct criterion without consecutive sessions to proceed during training, our crows had to maintain 75% correct over five consecutive sessions to advance, potentially enhancing the crows’ attention to stimulus details during training. Second, we used more pronounced deviations in quadrilaterals for the crows by displacing the bottom right vertex by 75% of the average vertex distance compared to 33% for the baboons. While these changes complicate direct performance comparisons between these two species (*10*), they have helped demonstrate that crows recognize geometric regularity, challenging the idea that intuitive shape geometry is uniquely human (*10*, *18*).

Spatial regularities are relevant for birds and other animals. Several studies indicate that birds use them for orientation and navigation in larger arenas (*14*, *15*, *16*). Pigeons have been shown to be sensitive to shape regularities of visual word forms (*25*). Some animal species are sensitive to symmetry in abstract patterns (*26*–*28*) or ecologically relevant sexual signals (*29*–*31*). However, while symmetry is just one aspect of defining the regularity of a two-dimensional shape, the angles and relative lengths of its sides are crucial features. Crows have been shown to discriminate the relationships of line lengths during categorical discrimination, potentially aiding their grasp of the varying side lengths in different quadrilateral shapes (*32*).

It has been argued that the geometric regularity effect in humans may stem from their ability to process embedded structures (*18*, *33*). Simple geometry of shapes often relies on nested or embedded spatial elements, such as lines and angles. In a parallelogram, for instance, sides are parallel and opposite sides are equal, forming nested relationships within the shape. Recognizing these relationships is crucial for identifying and categorizing types of quadrilaterals. Crows also demonstrate a rudimentary understanding of nested structures (*21*), suggesting their ability to grasp geometric properties in two-dimensional shapes. This basic intuition in crows exemplifies how core knowledge of magnitudes and geometry is rooted in biological evolution (*16*).

## MATERIALS AND METHODS

All procedures adhered to the National Institutes of Health’s Guide for the Care and Use of Laboratory Animals and were approved by the ethics committee and under license ZP 03/20 G by the national authority (Regierungspräsidium).

### Animals

Two male carrion crows (*Corvus corone corone*, crow 1: 11 years old, crow 2: 10 years old) were involved in this experiment. They were housed in social groups in large indoor aviaries. Throughout the training and testing periods, the crows were maintained on a controlled feeding protocol, and their weight was monitored daily. The crows earned their food during sessions and were fed afterward if necessary. Water was freely available ad libitum both in the aviary and during sessions.

### Experimental apparatus

The crows were positioned on a wooden perch inside an operant conditioning chamber equipped with a touchscreen (3 M. Microtouch, 15″, 60-Hz refresh rate) for presenting stimuli and detecting responses. The task sequence was controlled, and the crows’ responses were recorded using the CORTEX program developed by the National Institute of Mental Health. Head position of the crows was monitored using an infrared light barrier and a reflector attached to the top of their heads. An automated feeder-dispensed bird seed pellets and mealworms (*Tenebrio molitor* larvae) as rewards for correct responses during the sessions.

### Behavioral protocol

The crows were trained to detect one outlier shape among six shapes presented simultaneously on a computer screen. They initiated a trial by moving their head close to the screen (viewing distance of 14 cm), which closed a light barrier upon the appearance of a go-stimulus (small white “o”). The crows were required to maintain this head position during a prestimulus phase lasting 200 ms, where the screen remained empty. Subsequently, the stimuli were presented in a two by three array. The crows had to withhold their response for the first 150 ms after the stimuli appeared; any premature head movement resulted in the trial being automatically terminated and discarded.

The crows indicated their response by pecking on the stimulus that they judged to be different from the other five. They were allowed to respond immediately upon making their decision and had a maximum of 6000 ms to examine the stimuli. A food reward was provided for every correct response, accompanied by positive acoustic feedback. In case of erroneous responses, the crows received negative acoustic and visual feedback, followed by a 1500-ms time-out period.

### Training and generalization procedure

Inspired by the training procedure used for the baboons (*10*), we first trained the crows on a set of 10 stimulus pairs that differed in shape, color, or pattern. Each stimulus in a pair was presented both as an intruder and as a base stimulus (canonical and swapped presentation formats). The stimuli were presented in blocks of 120 conditions, with all stimuli in a block shown in a pseudo-random order. The number of trials per stimulus pair and presentation format was balanced within a block. The crows had to correctly answer each condition once to move on to the next block.

The crows were advanced to subsequent generalization phases when they achieved an average performance of at least 75% correct per session over a minimum of five consecutive sessions. In the first generalization phase, the crows were presented with 10 novel stimuli that differed in shape, color, or pattern, similar to the initial training. In the second generalization phase, they were presented with three pairs of geometric shapes. All stimuli used for training and generalization phases were randomly scaled in size (continuous scaling factor: 0.8 to 1.2) and randomly rotated (continuous angles: ± 25°). For each session, six independently scaled and rotated images were generated.

### Test stimuli and procedure

To test for the geometric regularity effect, we presented the crows with five pairs of quadrilateral shapes and their deviants (“intruders”) as probe stimuli. In addition, the test stimulus set contained five pairs of familiar, nonquadrilateral shapes. These background stimuli were included to keep the crows engaged and to counteract possible frustration from repeated failures with difficult probe stimuli. All stimuli in the test set were scaled in size (scaling factors: 0.875, 0.925, 0.975, 1.025, 1.075, and 1.125) and rotated (angles: ±5°, ±15°, and ±25°), chosen randomly from fixed values. The rotation and scaling factors used were identical to those for stimuli used with humans and baboons (*10*).

For each session, six independently scaled and rotated shapes were generated. The stimuli were presented in blocks of 120 conditions. Each block contained half background trials with nonquadrilateral shapes and half probe trials with quadrilateral shapes. Background and probe trials were randomly interleaved. In each block, each shape was presented once at all six positions, in both canonical and swapped presentation formats. The reference quadrilateral shapes were always compared to the same deviant shape within one block, with deviant shapes pseudorandomly changing over successive blocks. The same deviant was never shown in two consecutive blocks, and the number of presentations for all four deviant shapes was balanced across sessions.

### Quadrilateral probe stimuli and deviant shapes

We selected five quadrilaterals—square, rhombus, isosceles trapezoid, right hinge, and an arbitrary irregular quadrilateral—as reference shapes. These quadrilaterals were created using the same dimensions as those previously used in studies with humans and baboons (*10*). The selected quadrilaterals differ continuously in their geometric properties, including parallelism, symmetry, perpendicularity, equal sides, and equal angles.

For each reference shape, we generated four deviant shapes. These deviant shapes differed from their respective reference shapes by the position of the bottom right vertex. The bottom right vertex was displaced by a fixed distance of 75% of the average distance between all vertices. For two of the deviants, the bottom edge retained its orientation but was either shortened (deviant 1) or elongated (deviant 2). For the other two deviants, the bottom edge did not change in length but was either rotated upward (deviant 3) or downward (deviant 4), with the bottom left vertex remaining in place.

### Data analysis

All analyses were conducted in MATLAB (version R2022a, MathWorks Inc.) using custom-written software, except for Page’s trend test which was conducted in Python 3.9.13. In figures and in the text, statistical errors indicate the SEM. The significance level for all statistical tests was set at α = 0.05.

To investigate whether the crows were able to generalize the concept of the intruder task to quadrilateral stimuli, we specifically analyzed their detection performance in the trials where they encountered the quadrilateral shapes for the first time. Each combination of the presentation format (canonical or swapped) and the position of the intruder was considered a novel presentation. All five quadrilateral shapes were pooled for this analysis, resulting in 60 trials with a novel stimulus combination (2 presentation formats × 6 positions × 5 quadrilateral shapes).

A one-sided binomial test was computed to compare the number of correct detections (hits) in these trials against a chance level of 16.7% (1:6), which would be expected if the crows were pecking randomly at one of the six shapes. For a more detailed understanding of the performance in the very first trials, we analyzed the first 10 trials with quadrilateral stimuli, which encompassed a random, but unbalanced, selection from all 60 novel stimulus combinations. In addition, the same analysis was conducted separately for the two presentation formats. We investigated whether the geometric regularity of the quadrilateral shapes influenced the detection performance of the crows. Therefore, the quadrilaterals were sorted by their regularity with the square being the most regular shape and the irregular quadrilateral being the most irregular shape. The order of the three intermediate quadrilaterals followed the empirical regularity of the shapes that was derived from the error rates of human participants (*10*). This order slightly deviates from the theoretical geometric regularity measure, which underestimates the perceived regularity of symmetric shapes. In our case, this means that the rhombus and the isosceles trapezoid swap their positions.

The detection performance per quadrilateral for each block was computed separately. As in the previous analysis, only trials with first presentations for each quadrilateral and position were considered. Canonical and swapped presentations were pooled for this analysis, resulting in 12 first trials per quadrilateral per block. A binomial test, using the overall number of successful trials across blocks, was conducted to test the performance for each quadrilateral against the chance level.

Next, we tested the distribution of the averaged detection performances per quadrilateral for normality with a Kolmogorov-Smirnov test. Because of the non-normal distribution of the performance data, a Friedman test was conducted to determine whether the average detection performance across blocks differed between the quadrilateral shapes.

We also investigated whether the crows detected the intruder more frequently when the quadrilateral shape was more regular. A nonparametric Page’s trend test was computed to test the hypothesis that the average detection performance followed a monotonic trend according to the expected difficulty of the quadrilateral stimuli.

Last, we analyzed the crows’ intruder detection performance over time. The total number of presentation blocks over the entire test period was bisected, with the first half labeled as early and the second half as late blocks. The performance for early and late blocks was computed separately to investigate whether detection performance depended on geometric regularity. As previously described, a Page’s trend test was computed separately for early and late blocks to test whether the performance followed a monotonic trend according to the expected stimulus difficulty. To investigate whether the crows’ detection performance for the same quadrilateral changed over repeated presentations, a Mann-Whitney *U* test was conducted between early and late blocks for each quadrilateral.

## References

[1] J. F. Cantlon, E. M. Brannon, Shared system for ordering small and large numbers in monkeys and humans. Psychol. Sci. 17, 401–406 (2006). 16683927 10.1111/j.1467-9280.2006.01719.x

[2] A. Nieder, *A Brain for Numbers: The Biology of the Number Instinct* (MIT Press, 2019).

[3] A. Nieder, The adaptive value of numerical competence. Trends Ecol. Evol. 35, 605–617 (2020). 32521244 10.1016/j.tree.2020.02.009

[4] V. Izard, E. S. Spelke, Development of sensitivity to geometry in visual forms. Hum. Evol. 23, 213–248 (2009). 21359132 PMC3045057

[5] M. R. Dillon, E. S. Spelke, Core geometry in perspective. Dev. Sci. 18, 894–908 (2015). 25441089 10.1111/desc.12266 PMC4529807

[6] M. Amalric, L. Wang, P. Pica, S. Figueira, M. Sigman, S. Dehaene, The language of geometry: Fast comprehension of geometrical primitives and rules in human adults and preschoolers. PLOS Comput. Biol. 13, e1005273 (2017). 28125595 10.1371/journal.pcbi.1005273 PMC5305265

[7] M. R. Dillon, V. Izard, E. S. Spelke, Infants’ sensitivity to shape changes in 2D visual forms. Inf. Dent. 25, 618–639 (2020). 10.1111/infa.12343 32857438

[8] S. Dehaene, V. Izard, P. Pica, E. Spelke, Core knowledge of geometry in an Amazonian indigene group. Science 311, 381–384 (2006). 16424341 10.1126/science.1121739

[9] V. Izard, P. Pica, E. S. Spelke, S. Dehaene, Flexible intuitions of Euclidean geometry in an Amazonian indigene group. Proc. Natl. Acad. Sci. U.S.A. 108, 9782–9787 (2011). 21606377 10.1073/pnas.1016686108 PMC3116380

[10] M. Sablé-Meyer, J. Fagot, S. Caparos, T. van Kerkoerle, M. Amalric, S. Dehaene, Sensitivity to geometric shape regularity in humans and baboons: A putative signature of human singularity. Proc. Natl. Acad. Sci. U.S.A. 118, e2023123118 (2021). 33846254 10.1073/pnas.2023123118 PMC8072260

[11] I. Biederman, X. Yue, J. Davidoff, Representation of shape in individuals from a culture with minimal exposure to regular, simple artifacts: Sensitivity to nonaccidental versus metric properties. Psychol. Sci. 20, 1437–1442 (2009). 19883490 10.1111/j.1467-9280.2009.02465.x

[12] C. S. Henshilwood, F. d’Errico, K. L. Van Niekerk, L. Dayet, A. Queffelec, L. Pollarolo, An abstract drawing from the 73,000-year-old levels at Blombos Cave, South Africa. Nature 562, 115–118 (2018). 30209394 10.1038/s41586-018-0514-3

[13] K. Cheng, N. S. Newcombe, Is there a geometric module for spatial orientation? Squaring theory and evidence. Psychon. Bull. Rev. 12, 1–23 (2005). 15945200 10.3758/bf03196346

[14] D. M. Kelly, C. Chiandetti, G. Vallortigara, Re-orienting in space: Do animals use global or local geometry strategies? Biol. Lett. 7, 372–375 (2011). 21159689 10.1098/rsbl.2010.1024 PMC3097861

[15] L. Tommasi, C. Chiandetti, T. Pecchia, V. A. Sovrano, G. Vallortigara, From natural geometry to spatial cognition. Neurosci. Biobehav. Rev. 36, 799–824 (2012). 22206900 10.1016/j.neubiorev.2011.12.007

[16] E. S. Spelke, S. A. Lee, Core systems of geometry in animal minds. Philos. Trans. R. Soc. Lond. B Biol. Sci. 367, 2784–2793 (2012). 22927577 10.1098/rstb.2012.0210 PMC3427547

[17] G. Westphal-Fitch, L. Huber, J. C. Gómez, W. T. Fitch, Production and perception rules underlying visual patterns: Effects of symmetry and hierarchy. Philos. Trans. R. Soc. Lond. B Biol. Sci. 367, 2007–2022 (2012). 22688636 10.1098/rstb.2012.0098 PMC3367690

[18] S. Dehaene, F. Al Roumi, Y. Lakretz, S. Planton, M. Sablé-Meyer, Symbols and mental programs: A hypothesis about human singularity. Trends Cogn. Sci. 26, 751–766 (2022). 35933289 10.1016/j.tics.2022.06.010

[19] L. Veit, A. Nieder, Abstract rule neurons in the endbrain support intelligent behaviour in corvid songbirds. Nat. Commun. *4*, 2878 (2013). 24285080 10.1038/ncomms3878

[20] A. Smirnova, Z. Zorina, T. Obozova, E. Wasserman, Crows spontaneously exhibit analogical reasoning. Curr. Biol. 25, 256–260 (2015). 25532894 10.1016/j.cub.2014.11.063

[21] D. A. Liao, K. F. Brecht, M. Johnston, A. Nieder, Recursive sequence generation in crows. Sci. Adv. 8, eabq3356 (2022). 36322648 10.1126/sciadv.abq3356 PMC9629703

[22] H. M. Ditz, A. Nieder, Format-dependent and format-independent representation of sequential and simultaneous numerosity in the crow endbrain. Nat. Commun. 11, 686 (2020). 32019934 10.1038/s41467-020-14519-2 PMC7000399

[23] M. E. Kirschhock, A. Nieder, Number selective sensorimotor neurons in the crow translate perceived numerosity into number of actions. Nat. Commun. 13, 6913 (2022). 36376297 10.1038/s41467-022-34457-5 PMC9663431

[24] D. A. Liao, K. F. Brecht, L. Veit, A. Nieder, Crows “count” the number of self-generated vocalizations. Science 384, 874–877 (2024). 38781375 10.1126/science.adl0984

[25] D. Scarf, K. Boy, A. Uber Reinert, J. Devine, O. Güntürkün, M. Colombo, Orthographic processing in pigeons (*Columba livia*). Proc. Natl. Acad. Sci. U.S.A. 113, 11272–11276 (2016). 27638211 10.1073/pnas.1607870113 PMC5056114

[26] J. D. Delius, B. Nowak, Visual symmetry recognition by pigeons. Psychol. Res. 44, 199–212 (1982). 7156265 10.1007/BF00308420

[27] M. Giurfa, B. Eichmann, R. Menzel, Symmetry perception in an insect. Nature 382, 458–461 (1996). 18610516 10.1038/382458a0

[28] E. Clara, L. Regolin, G. Vallortigara, Preference for symmetry is experience dependent in newborn chicks (*Gallus gallus*). J. Exp.Psychol. Anim. Behav. Process. 33, 12–20 (2007). 17227191 10.1037/0097-7403.33.1.12

[29] A. P. Møller, Female swallow preference for symmetrical male sexual ornaments. Nature 357, 238–240 (1992). 1589021 10.1038/357238a0

[30] J. P. Swaddle, I. C. Cuthill, Preference for symmetric males by female zebra finches. Nature 367, 165–166 (1994).

[31] M. R. Morris, K. Casey, Female swordtail fish prefer symmetrical sexual signal. Anim. Behav. 55, 33–39 (1998). 9480669 10.1006/anbe.1997.0580

[32] L. Wagener, A. Nieder, Categorical representation of abstract spatial magnitudes in the executive telencephalon of crows. Curr. Biol. 33, 2151–2162.e5 (2023). 37137309 10.1016/j.cub.2023.04.013

[33] M. Sablé-Meyer, K. Ellis, J. Tenenbaum, S. Dehaene, A language of thought for the mental representation of geometric shapes. Cogn. Psychol. 139, 101527 (2022). 36403385 10.1016/j.cogpsych.2022.101527

