# Supplementary material for: Crows recognize geometric regularity
Source: Sci Adv. 2025 Apr 11;11(15):eadt3718. doi: 10.1126/sciadv.adt3718 (PMC11988402; doi:10.1126/sciadv.adt3718)
Supplement: Supplementary file 1 — Figs. S1 to S4 Table S1 [file sciadv.adt3718_sm.pdf]

Science Advances
AAAS

Supplementary Materials for

Crows recognize geometric regularity

Philipp Schmidbauer *et al.*

Corresponding author: Andreas Nieder, andreas.nieder@uni-tuebingen.de

Sci. Adv. **11**, eadt3718 (2025)
DOI: 10.1126/sciadv.adt3718

This PDF file includes:

Figs. S1 to S4
Table S1

Supplementary Figures

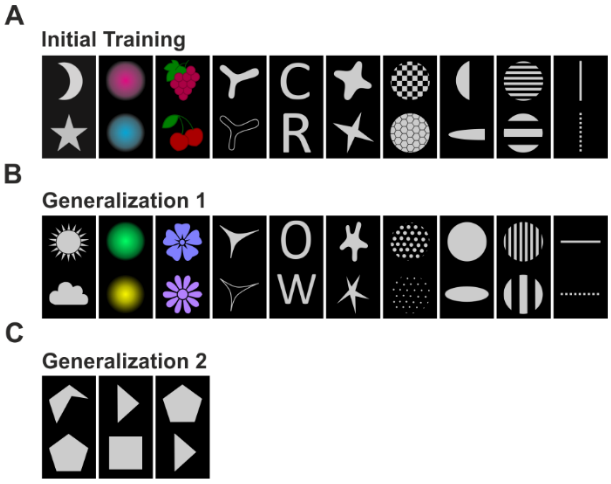

Supplementary Figure 1. Stimulus pairs used during the training and generalization phases. **A)** Initial training stimuli: Crows were trained to detect the intruder using ten stimulus pairs. The stimuli in each pair differed in color, shape, or pattern. **B)** Generalization 1 stimuli: Similar to the initial training, the stimuli in each pair differed in color, shape, or pattern. **C)** Generalization 2 stimuli: In the second generalization phase, stimulus pairs differed in their geometric shape. Note that no stimulus pair ever encompassed two quadrilateral shapes.

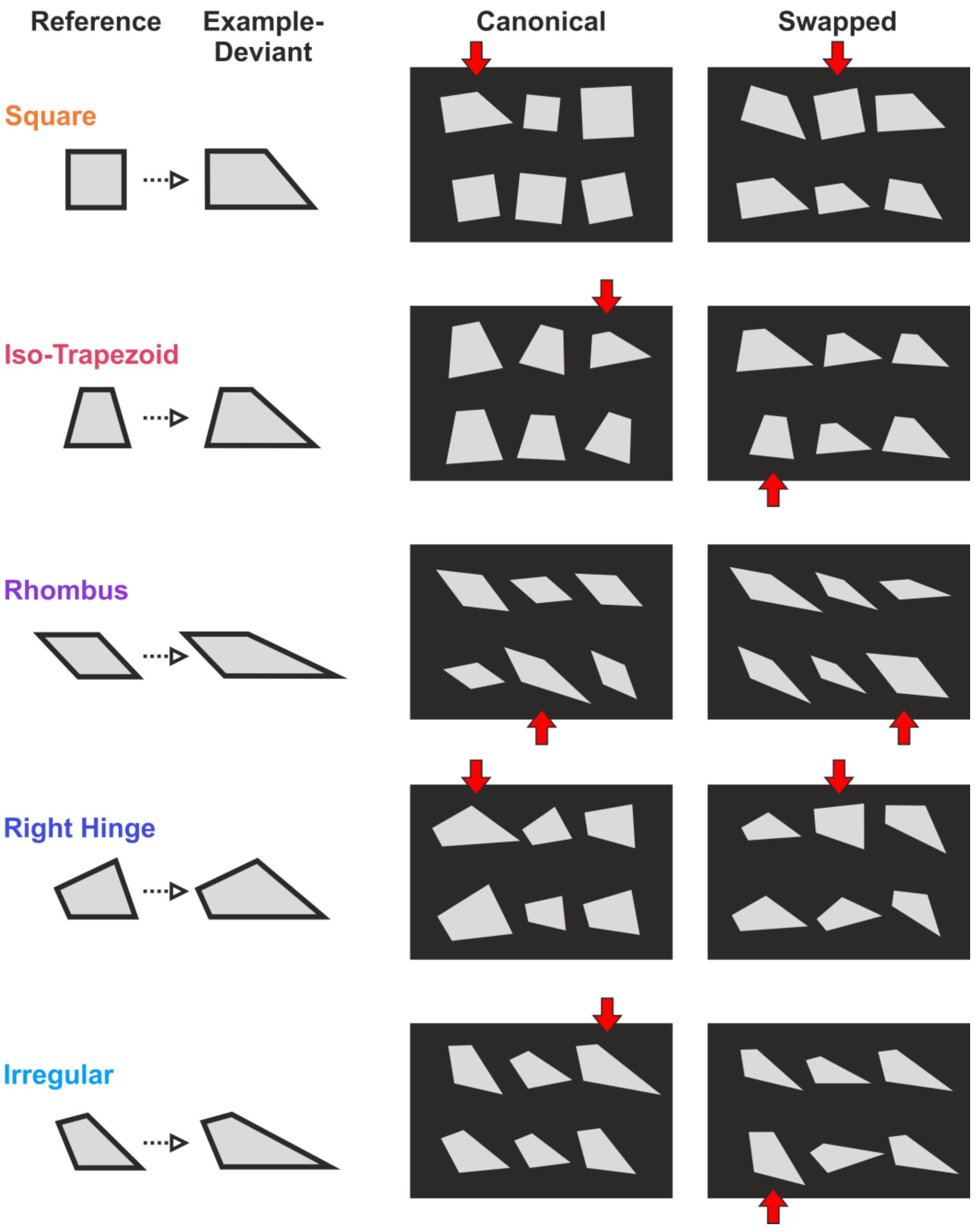

Supplementary Figure 2: Examples of quadrilateral probe stimulus layouts. From the five used reference shapes (square, iso-trapezoid, rhombus, right hinge, irregular), four deviant shapes were generated (method depicted in **Figure 2C**) that differed in geometric regularity from the reference (only one deviant shape shown). Detection of the deviant shape was tested in two different formats: in the 'canonical' presentation format, one randomly-placed deviant shape had to be recognized as the intruder (indicated by red arrow) amidst five reference shapes; in the 'swapped' presentation format, the reference shape was the intruder and had to be detected amidst five deviant shapes.

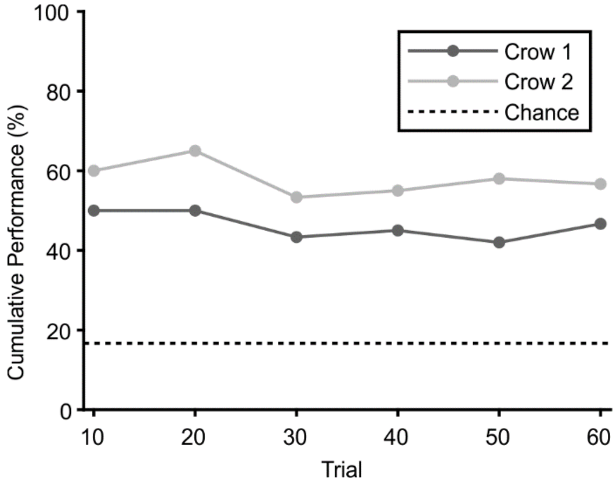

Supplementary Figure 3. Cumulative performance from the first 10 to the first 60 probe trials. Each dot represents cumulative performance, i.e., the performance across all trials from the first trial up to the respective trial number on the x-axis.

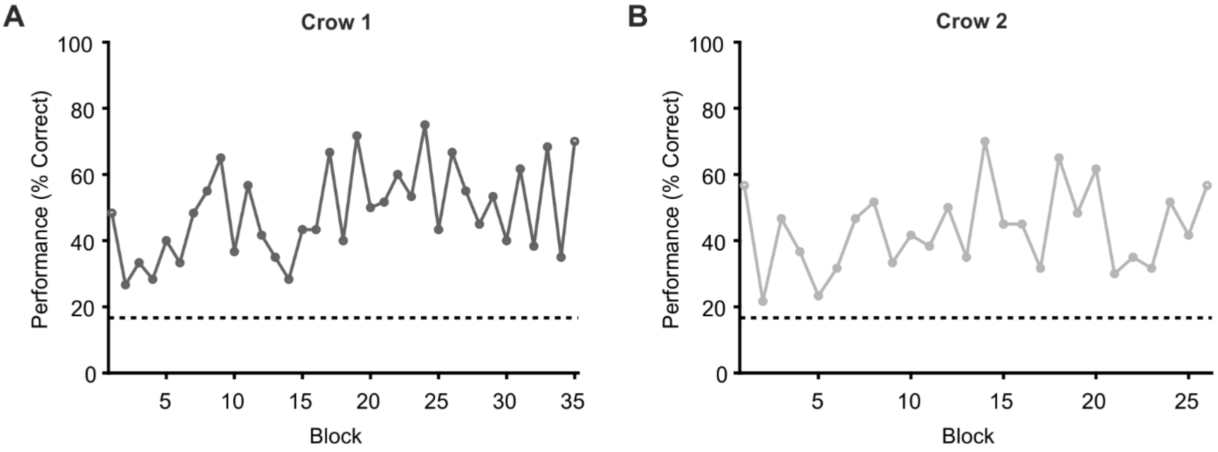

Supplementary Figure 4. Probe trial performance per blocks and sessions. **A)** Average performance of crow 1 per block and session for quadrilateral probe stimuli only. The dashed line indicates the chance level. **B)** Average performance of crow 2. Same layout as in panel A.

Supplementary Tables

Supplementary Table 1. Definition of the five quadrilateral reference shapes.

Quadrilateral	Vertex coordinates				Shape parameters		
	Bottom left	Bottom right	Top left	Top right	Perimeter	Area	Geometric Properties
Square	(0, 0)	(1.26, 0)	(0, 1.26)	(1.26, 1.26)	1	1	19
Rhombus	(0, 0)	(1.3, 0)	(-0.908, 0.931)	(0.392, 0.931)	1.032	0.762	9
Iso-trapezoid	(0, 0)	(1.5, 0)	(0.365, 1.362)	(1.109, 1.362)	1.006	0.962	5
Right hinge	(0, 0)	(1.5, 0)	(-0.296, 0.634)	(1.064, 1.268)	1	0.929	2
Irregular	(0, 0)	(1.5, 0)	(-0.45, 1.058)	(0.227, 1.24)	1.017	0.836	0

The precise shape of each quadrilateral was defined by the Cartesian coordinates (x, y) of its four vertices. We exactly reproduced five of the eleven quadrilaterals that were previously used to test humans and baboons on their sensitivity to geometric regularity (Sablé-Meyer et al., 2021, Supplementary Information, Table S1). All shapes had the same average distance of 1.434 units between all pairs of vertices. Due to the limited degrees of freedom when constructing these specific quadrilaterals, the perimeter and the area were not matched between shapes. The perimeter and area values were normalized relative to the respective values of the square. The last column shows the number of geometric properties for each shape. This value results from the count of parallel lines, symmetries, right angles, equal sides, and equal angles that occur in the respective shape. For example, the five geometric properties of the isosceles trapezoid result from one pair of parallel lines, one symmetry axis, no right angles, one pair of lines with equal length, and two pairs of equal angles.
